# Elevated postprandial triglyceride-rich lipoproteins in patients with diabetes and stable coronary artery disease correlated with early renal damage and systemic inflammation

**DOI:** 10.1186/s12944-023-01820-4

**Published:** 2023-05-03

**Authors:** Xu Guo, Yujia Zhai, Chenliang Song, Zhen Mi, Jiya Peng, Jing Guo, Xianzhuo Teng, Daqing Zhang

**Affiliations:** 1 Department of Cardiology, Shengjing Hospital of China Medical University, #36 Sanhao Street, Heping District, Shenyang City, Liaoning Province 110004 People’s Republic of China; 2 Department of Critical Care Medicine, Hainan General Hospital, Haikou, China

**Keywords:** Stable coronary artery disease, Postprandial dyslipidaemia, Urinary albumin to creatinine ratio, Diabetic mellitus

## Abstract

**Background:**

Dyslipidaemia is key in the development of coronary heart disease (CHD) in patients with diabetes mellitus (DM). Accumulated evidence supports that diabetic nephropathy increases the mortality risk of patients with CHD, while the influence of diabetic dyslipidaemia on renal damage in patients with DM and CHD remains unknown. Moreover, recent data indicate that postprandial dyslipidaemia has predictive value in terms of CHD prognosis, especially in patients with DM. The study aimed to determine the relationship of triglyceride-rich lipoproteins (TRLs) after daily Chinese breakfast on systemic inflammation and early renal damage in Chinese patients with DM and SCAD.

**Methods:**

Patients with DM diagnosed with SCAD while in the Department of Cardiology of Shengjing Hospital from September 2016 to February 2017 were enrolled in this study. Fasting and 4-h postprandial blood lipids, fasting blood glucose, glycated haemoglobin, urinary albumin-to-creatinine ratio (UACR), serum interleukin-6 (IL-6) and tumour necrosis factor-α (TNF-α) concentrations, and other parameters were measured. Fasting and postprandial blood lipid profiles and inflammatory cytokines were analysed using a paired t-test. The association between variables was analysed using Pearson or Spearman bivariate analysis. *P* < 0.05 was considered to be statistically significant.

**Results:**

The study enrolled 44 patients in total. Compared with fasting state, postprandial total cholesterol high-density lipoprotein-cholesterol (HDL-C),low-density lipoprotein-cholesterol (LDL-C) and non-high-density lipoprotein-cholesterol (non-HDL-C) all showed no significant change. Postprandial serum triglyceride (TG) concentration increased significantly compared with that at fasting (1.40 ± 0.40 vs. 2.10 ± 0.94 mmol/L, *P* < 0.001), as did serum remnant lipoprotein-cholesterol (RLP-C) (0.54 ± 0.18 mmol/L vs. 0.64 ± 0.25 mmol/L). Pearson analysis revealed that serum TG and RLP-C positively correlated before and after breakfast. Moreover, during fasting, positive correlations were observed between TG and serum IL-6, TNF-α, and UACR. Positive correlations were observed between RLP-C and IL-6, UACR under fasting condition, while both TG and RLP-C were positively correlated with postprandial serum IL-6, TNF-α, and UACR concentrations. Finally, positive correlations were observed between UACR and IL-6 and TNF-α concentration under both fasting and postprandial conditions.

**Conclusions:**

An increase in postprandial TRLs was observed in Chinese patients with DM and SCAD after daily breakfast, and this increase may be related to early renal injury via the induction of systemic inflammation.

**Supplementary Information:**

The online version contains supplementary material available at 10.1186/s12944-023-01820-4.

## Background

Diabetes mellitus (DM) is an independent risk factor for coronary heart disease (CHD) [1]. Furthermore, dyslipidaemia is crucial in the development and prognosis of CHD in patients with DM. Despite recent evidence indicating that diabetic nephropathy, significantly increases the death rate of patients with CHD [2], the influence of diabetic dyslipidaemia on renal damage in patients with DM and CHD remains unknown. Diabetic dyslipidaemia is characterised by hypertriglyceridaemia (HTG), decreased high density lipoprotein-cholesterol (HDL-C), and increased small dense low-density lipoprotein (sdLDL) particles [3]. While recent evidence has shown that HTG worsens the prognosis of cardiovascular disease [4], it does not appear to directly promote the atherosclerotic process. Clinically, HTG represents enhanced levels of serum chylomicron (CM), very low-density lipoprotein (VLDL), and intermediate density lipoprotein (IDL). All of them belong to Triglyceride-rich lipoproteins (TRLs). During lipolysis, lipoprotein lipase and hepatic lipase degrade TRLs into remnant-like particles (RLPs), which are subsequently enriched with cholesterol (RLP-C). Importantly, elevated RLP-C contributes to the development of atherosclerotic cardiovascular diseases (ASCVDs) [5].

Assessment of dyslipidaemia conventionally requires individuals to fast for 8–12 h [6]. However, as we spend most of our lives in a state of non-fasting, testing while fasting may not be physiologically accurate. Moreover, compared with LDL-C, TRL level is more significantly affected by diet, and as such, observable increases in postprandial TRLs have more predictive value for CHD morbidity and mortality, especially in patients with DM [7, 8]. Previous studies of patients with DM have indicated that enhanced TRLs following high-fat meals induce systemic inflammation, with an increased level of serum interleukin-6 (IL-6) and tumour necrosis factor-α (TNF-α) [9, 10]. Furthermore, it has been demonstrated that this systemic inflammation contributed significantly to renal damage in patients with DM [11]. The influence of Chinese daily diet on postprandial dyslipidaemia and relevant systemic inflammation in patients with DM remains unclear. Importantly however, it has yet to be determined if postprandial TRLs are related to renal damage in patients with DM and CHD. As such, the study aimed to investigate the impact of triglyceride-rich lipoproteins (TRLs) after daily Chinese breakfast on systemic inflammation and renal damage in Chinese patients with DM and SCAD.

Moreover, considering TG level at 4 h after a meal has good repeatability [9], we adopted 4 h after daily breakfast as the entry point to observe postprandial lipid profile and inflammation cytokines in this study.

## Methods

### Participants

Participants in this study were patients with DM who were hospitalised or treated as an outpatient with SCAD at the Department of Cardiology, Shengjing Hospital, China Medical University from September 2016 to February 2017. Each patient underwent careful evaluation by two cardiologists to determine if they met the inclusion criteria. For in-hospital patients, the reason for hospital admission was also documented.

#### Inclusion criteria

The diagnostic criteria for SCAD were based on the 2013 ESC guideline and 2007 Chinese guideline [12, 13], SCAD generally refers to transient ischemic chest discomfort, which is usually induced by exercise, emotion or other stress and reproducible, occasionally occurring spontaneously. SCAD is commonly caused by episodes of reversible myocardial demand/supply mismatch, which also includes the stabilized, often asymptomatic, phases after an acute coronary syndrome. Patients with DM were diagnosed based on the World Health Organisation guidelines (1999).

#### Exclusion criteria

Patients meeting any of the following criteria were excluded: acute coronary syndromes; NYHA III–IV; various malignant tumours, pulmonary tuberculosis, severe trauma, burns, systemic lupus erythematosus, chronic suppurative infection, chronic blood loss and other diseases that consume excessive energy substances of the body and cause negative energy balance of the body, gastrointestinal or pancreatic diseases which affect food and drug absorption, thyroid dysfunction, abnormal liver (ALT > 40 U/ L) or estimated glomerular filtration rate (eGFR) < 60 ml/ min/ 1.73 m^2^, familial hypercholesterolaemia, fasting TG level > 5.6 mmol/L, history of major surgery diagnosis of an infectious disease in the previous 6 months, or routine use of glucocorticoids or immunosuppressive agents.

### Patient demographic data

The demographic data for all patients enrolled in this study was collected, including age, sex, weight, history of hypertension, smoking, use of lipid-lowering drug, and myocardial infarction.

### Collection of blood samples

Venous blood samples (5 mL) were obtained daily at two timepoints: (1) fasting (0-h) and (2) 4 h (4-h) following breakfast. Fasting blood collection required the patient to abstain from food beginning at 8 pm the previous night. The postprandial blood samples were collected at 4 h after the first bite of daily breakfast between 7–8 am. Patients were required to fast, but there were no limitations on water consumption; additionally, the patients were advised to avoid intense exercise during the period.

### Biochemical profiles

All biochemical indicators were measured at the laboratory centre within our hospital. The levels of plasma and urine creatinine (Cr) were assessed via a RANDOX enzymatic assay, FBG was measured using the hexokinase method. The levels of plasma TC was measured bycholesterol oxidase method (TC test kit, Kyowa Medex Co.,Ltd.). Serum TG was detected by GB&Enzyme Method (TG test kit, Kyowa Medex Co.,Ltd.). The level of HDL-C was determined using the chemical modification enzyme method (HDL-C test kit, Kyowa Medex Co.,Ltd.) and plasma LDL-C levels were detected using a selective solubilisation method (LDL-C test kit, Kyowa Medex Co.,Ltd.). Chemiluminescence microparticle immunoassay (competitive inhibition method) was used for the detection of plasma free triiodothyronine (FT3) and free thyroxine (FT4) levels, and chemiluminescence microparticle immunoassay (sandwich method) was used to determine plasma thyroid-stimulating hormone (TSH) levels. Plasma aspartate transaminase (AST) and alanine transaminase (ALT) concentrations were detected using the reduced coenzyme NADH method. All the above mentioned biochemical indicators were detected using an Abbott ARCHITECT ci16200 biochemical immunoassay. Glycated haemoglobin (HbA1c) level was detected using the Bio-RAD VARIANT II HbA1c analyser via liquid chromatography. Urinary albumin was measured on an Immage800 Immunoturbator using rate turbidimetry. Ratio of urinary albumin to creatinine (UACR) was calculated as follows: UACR = urinary albumin × 1000/ (urine Cr × 0.113). The patient’s eGFR was calculated according to the Cockcroft-Gault formula $[\mathrm{eGFR }= (140-\mathrm{age}) \times \mathrm{ body weight}/ 0.818 *\mathrm{ Cr }(\mathrm{\mu mol}/\mathrm{ L}),\mathrm{ calculated results}* 0.85\mathrm{ for female}].$

### Calculation formula

RLP-C and non-HDL-C concentrations were calculated using the formulas below:

$$\mathrm{RLP}-\mathrm{C}=\mathrm{TC}-\left(\mathrm{LDL}-\mathrm{C}+\mathrm{HDL}-\mathrm{C}\right)$$

$$\mathrm{non}-\mathrm{HDL}-\mathrm{C}=\mathrm{TC}-\mathrm{HDL}-\mathrm{C}$$

### Detection of inflammatory cytokines

Fasting (0-h) and 4-h postprandial serum was centrifuged (3500 r/min) at 4 °C on the same day of biochemical detection. Serum was separated and stored at -80 °C for subsequent use. Serum IL-6 and TNF-α concentrations were detected using an enzyme-linked immunosorbent assay (ELISA) kit (Thermo Fisher Scientific, Australia). Absorbance was detected at 450 nm using a microplate reader (BioTek Instruments, USA). The analytical sensitivity of the assay was set at < 2 pg/mL for human IL-6 and < 1.7 pg/ mL for human TNF-α.

### Statistical analysis

SPSS 22 software was used in the study. Normally distributed data are expressed as mean ± standard deviation (SD), and skewness data are expressed as median (lower quartile, upper quartile). The count data are expressed as number of cases (percentage). Blood lipid profile and inflammatory cytokines at fasting (0-h) and 4-h postprandial were analysed using paired t-test. The correlation between variables was analysed using Pearson or Spearman bivariate correlation analysis. Statistically significance was set at *P* < 0.05.

## Results

### Patients’ baseline characteristics

Forty-four patients with SCAD and DM were enrolled in this study, including 25 males and 19 females. Of these patients, 34 were from the outpatient department, while the remaining ten patients were in-patients. As the results in Table 1 show, the average patient age was 64.1 ± 8.9 years, with an average body weight of 73.8 ± 11.3 kg and an average eGFR of 90.23 ± 22.27 ml/ min. Patients with a history of hypertension, smoking, use of lipid-lowering drugs or history of myocardial infarction accounted for 84.1%, 34.1%, 95.5%, and 15.9% of all patients, respectively. The levels of HbA1c, FBG, and UACR were 6.90 (5.70–12.40) %, 6.75 (3.38–14.96) mmol/L, and 7.70 (2.40–102.10) mg/g, respectively.

**Table 1:** Demographic characteristics of patients with DM and SCAD

	*N* = 44
Age, years	64.1 ± 8.9^b^
Sex, male/female	25/19^a^
Weight, kg	73.8 ± 11.3^b^
Hypertension, cases (%)	37 (84.1%)^a^
Smoker, cases (%)	15 (34.1%)^a^
Myocardial infarction, cases (%)	7 (15.9%)^a^
Lipid-lowering drugs, cases (%)	42 (95.5%)^a^
Insulin, cases (%)	12 (27.2%)
Duration of diabetes (years)	5 (1–23)
HbA1c (%)	6.90 (5.70–12.40)^c^
FBG (mmol/L)	6.75 (3.38–14.96)^c^
eGFR (ml/min)	90.23 ± 22.27^b^
UACR (mg/g)	7.70 (2.40–102.10)^c^
ALT (U/L)	19.98 ± 17.24^b^
AST (U/L)	17.16 ± 4.72^b^
TSH (uIU/1L)	1.99 ± 1.96^b^
FT4 (p1ol/L)	13.47 ± 1.88^b^
FT3 (p1ol/L)	3.91 ± 0.77^b^

*Abbreviations*: *HbA1c* glycated haemoglobin; *FBG* fasting blood glucose, *eGFR* estimated glomerular filtration rate, *UACR* ratio of urinary albumin to creatinine, *SCAD* stable coronary artery disease, *AST* aspartate transaminase, *ALT* alanine transaminase, *FT4* free thyroxine, *FT3* triiodothyronine, *TSH* thyroid-stimulating hormone

^a^data expressed as number of cases (percentage)

^b^normal distribution data expressed as mean ± standard deviation

^c^skewness expressed as median (lower quartile, upper quartile)

### Serum lipids at fasting and 4-h postprandial

The results of the serum lipid concentration analyses are shown in Table 2. In comparison with the fasting state, 4-h postprandial TC, LDL-C, and HDL-C concentrations did not show any significant change. Similarly, the calculated non-HDL-C concentration was not significantly different under fasting conditions compared with 4-h postprandial. In contrast, the TG concentration at 4-h postprandial was significantly higher than at fasting with an average increase of 0.69 mmol/L. Moreover, the 4-h postprandial serum RLP-C concentration was significantly higher than that measured under fasting conditions.

**Table 2:** Comparison analysis of blood lipid indexes between fasting and 4-h postprandial condition (mmol/L)

Indexes	0-h	4-h	Δ,(4-h–0-h)	^*^P
TC	3.91 ± 0.98	3.87 ± 0.97	-0.04 ± 0.64	0.651^b^
LDL-C	2.40 ± 0.83	2.25 ± 0.80	-0.14 ± 0.58	0.123^b^
HDL-C	0.98 ± 0.28	0.96 ± 0.26	-0.01 ± 0.17	0.634^b^
TG	1.40 ± 0.40	2.10 ± 0.94	0.69 ± 0.83	< 0.01^b^
RLP-C	0.54 ± 0.18	0.64 ± 0.25	0.11 ± 0.22	0.002^b^
Non-HDL-C	2.93 ± 0.89	2.90 ± 0.92	-0.03 ± 0.65	0.751^b^

*Abbreviations*: *TC* total cholesterol, *LDL-C* low-density lipoprotein-cholesterol, *HDL-C* high-density lipoprotein-cholesterol, *TG* triglyceride, *RLP-C* remnant lipoprotein-cholesterol, *non-HDL-C* non-high-dense lipoprotein-cholesterol, *h* hour, *0-h* fasting condition, Δ the difference between 4 h post-prandial and fasting condition

^*^P comparison of fasting and postprandial blood lipids by paired T test

^b^normal distribution data

### Associations between serum TG, RLP-C and non-HDL-C concentrations

**Fig. 1:**
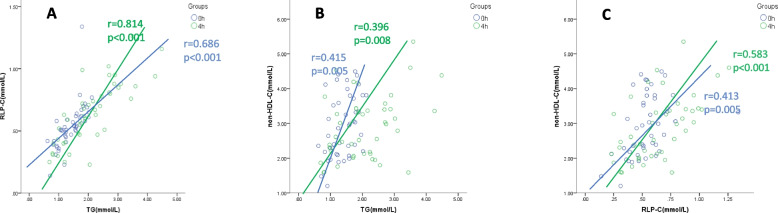
Correlation analysis of serum TG, RLP-C, and non-HDL-C concentrations under fasting and postprandial conditions. Pearson correlation analysis shows positive correlation among between TG, RLP-C and non-HDL-C. RLP-C is more closely correlated with TG/non-HDL-C at 4-h postprandial. TG is more closely correlated to non-HDL-C under the fasting condition. Bivariate correlation analysis, r represents the Pearson coefficient of correlation. Abbreviations: TG, triglyceride; RLP-C, remnant lipoprotein-cholesterol; non-HDL-C, non-high-density lipoprotein-cholesterol

Pearson correlation analysis showed that serum TG positively correlated with RLP-C under both fasting (*r* = 0.686, *P* < 0.01, Fig. 1A) and 4-h postprandial conditions (*r* = 0.814, *P* < 0.01, Fig. 1A). Importantly, the correlation was stronger at 4-h postprandial. In addition to this, serum TG concentration was also positively correlated with non-HDL-C under both fasting (*r* = 0.415, *P* = 0.005, Fig. 1B) and 4-h postprandial conditions (*r* = 0.396, *P* = 0.008, Fig. 1B). RLP-C positively correlated with non-HDL-C under fasting (*r* = 0.413, *P* = 0.005, Fig. 1C) and 4-h postprandial conditions (*r* = 0.583,* P* = 0.001, Fig. 1C).

### Systemic inflammation markers at fasting and 4-h postprandial

Serum concentrations of IL-6 (15.40 ± 1.39 vs. 14.72 ± 1.35 pg/ml, *P* = 0.002) and TNF-α (24.39 ± 2.40 vs. 23.41 ± 1.50 pg/ml, *P* = 0.007) were significantly increased at 4-h postprandial compared with the fasting state, at 0.69 ± 1.40 pg/ml and 0.98 ± 2.20 pg/ml, respectively.

### Correlation between blood lipid parameters and inflammation markers at fasting and 4-h postprandial

**Fig. 2:**
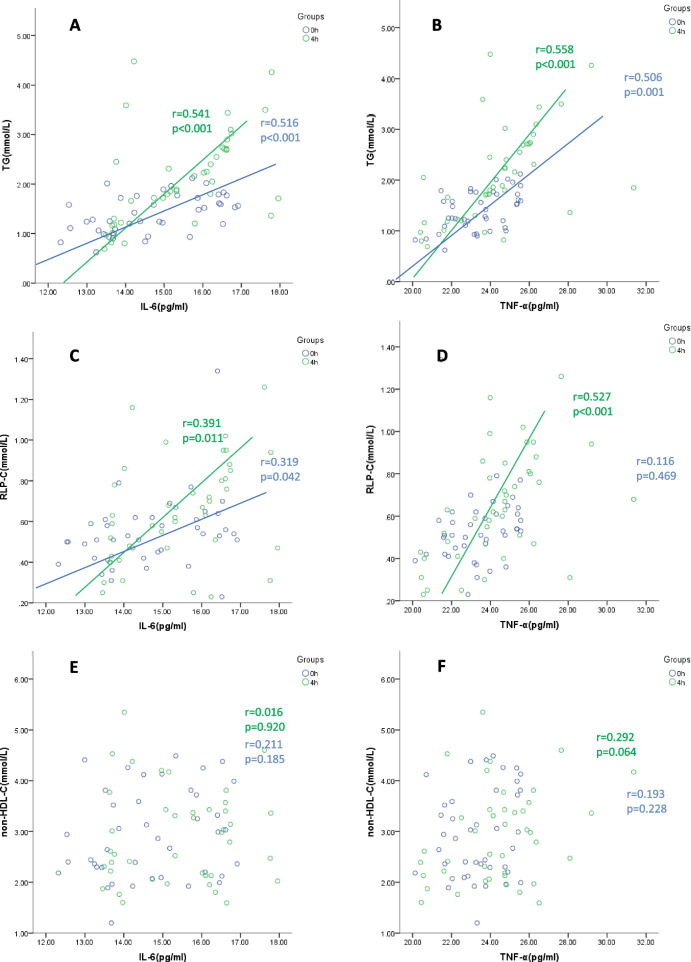
TRL concentrations correlated with systemic inflammation under fasting and postprandial conditions in patients with DM and SCAD. Pearson correlation analysis shows TRLs (TG/RLP-C) and systemic inflammation markers (IL-6/TNF-α) are positively correlated under both fasting and postprandial conditions, with the correlation stronger in the postprandial conditions. Serum TNF-α concentration is only correlated with RLP-C under the postprandial condition. Bivariate correlation analysis, r represents the Pearson coefficient of correlation. Abbreviations: IL-6, interleukin-6; TNF-α, tumour necrosis factor-α; TG, triglyceride; RLP-C, remnant lipoprotein-cholesterol; non-HDL-C, non-high-density lipoprotein-cholesterol

Pearson analysis showed a positive correlation between serum TG and IL-6 concentrations at fasting and 4-h postprandial (Fig. 2A), with similar correlation also observed between serum TG and TNF-α concentration (Fig. 2D). Serum RLP-C and IL-6 concentrations were positively correlated under fasting (*r* = 0.319, *P* = 0.042) and 4-h postprandial (*r* = 0.391, *P* = 0.011) conditions (Fig. 2B). Interestingly, serum RLP-C concentration was only positively correlated with serum TNF-α concentration at 4-h postprandial (Fig. 2E). No other lipid showed any significant correlation with inflammation markers.

### Correlation between blood lipid parameters and UACR at fasting and 4-h postprandial

**Fig. 3:**
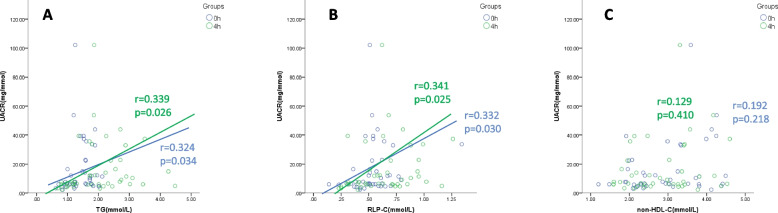
UACR is correlated with serum TRL level concentrations in patients with DM and SCAD. Spearman correlation analysis shows that UACR and blood TG/RLP-C concentrations are positively correlated under both fasting and postprandial conditions, with the correlation stronger in the postprandial condition. UACR has no correlation with non-HDL-C under either the fasting or postprandial condition. Bivariate correlation analysis, r represents the Spearman coefficient of correlation. Abbreviations: TG, triglyceride; RLP-C, remnant lipoprotein-cholesterol; non-HDL-C, non-high-dense lipoprotein-cholesterol; UACR, ratio of urinary albumin to creatinine; TRLs, triglyceride-rich lipoproteins

Spearman correlation analyses revealed significant positive correlations between UACR and serum TG (fasting state: *r* = 0.324, *P* = 0.034; postprandial state: *r* = 0.339, *P* = 0.026) and RLP-C (fasting state: *r* = 0.332, *P* = 0.030; postprandial state: *r* = 0.341, *P* = 0.025) concentration, observed under both fasting and 4-h postprandial conditions, with the correlation coefficient increased at 4-h postprandial (Fig. 3A, B). No correlation was observed between other lipid parameters and UACR (Fig. 3C).

### Correlation between serum inflammation markers and UACR at fasting and 4-h postprandial

**Fig. 4:**
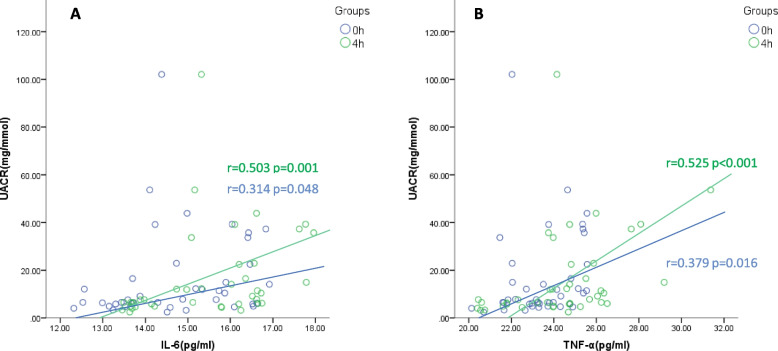
UACR is correlated with systemic inflammation markers in patients with DM and SCAD. Spearman correlation analysis shows UACR and serum IL-6/TNF concentrations are positively correlated under both fasting and postprandial conditions, with the correlation stronger in the postprandial condition. Bivariate correlation analysis, r represents the Spearman coefficient of correlation. Abbreviations: IL-6, interleukin-6; TNF-α, tumour necrosis factor-α; UACR, ratio of urinary albumin to creatinine

Spearman correlation analyses revealed a weak correlation between serum IL-6 concentration and UACR under fasting conditions (*r* = 0.314, *P* = 0.048). However, the correlation was much stronger at 4-h postprandial (*r* = 0.503, *P* = 0.001) (Fig. 4A). Serum TNF-α concentration at 4-h postprandial (*r* = 0.525, *P* < 0.001) was consistently more positively correlated with UACR compared with at fasting (*r* = 0.379, *P* = 0.016) (Fig. 4B).

## Discussion

Dyslipidaemia accelerates ASCVD in patients with DM. According to research, non-fasting blood lipid tests indicate the lipid status of DM patients more comprehensively than when measured during fasting, and as such exhibit greater prognostic value for cardiovascular risk [14]. Diabetic nephropathy significantly affects the prognosis of patients with CHD and DM [15]. In this study, UCAR was used an early marker for diabetic nephrology to explore the influence and potential mechanism of postprandial lipids on renal damage in patients with DM and SCAD.

This study further verified cholesterol parameters (TC, LDL-C and HDL-C) were not significantly different in fasting and non-fasting conditions, indicating that these cholesterol-related parameters are not affected by Chinese food intake in DM patients with SCAD. Non-HDL-C, represents all potentially atherogenic lipoprotein particles and reports have demonstrated that it is not affected by daily Chinese breakfast [16]. Considering the consistent concentrations of LDL-C and non-HDL-C between the fasting and 4-h postprandial conditions, postprandial blood collection could avoid hypoglycaemia in DM patients; therefore, it is reasonable to use postprandial lipid testing to evaluate cholesterol in Chinese CHD patients, especially those with DM.

The most significant difference observed in this study between fasting and postprandial lipid concentrations was primarily in TG and RLP-C, which is consistent with previous studies that have demonstrated that high-fat meals increase serum concentrations of TG and RLP-C [17, 18]. Furthermore, as the acute inhibitory effect insulin has on TRL production under the postprandial condition is reduced in type 2 diabetes, the extent and duration of elevated postprandial HTG should be significantly increased in DM patients compared to those without DM. This was confirmed as we demonstrated that serum TG and RLP-C levels were more pronounced in the postprandial condition in DM patients with SCAD. In light of this, the metabolic mechanisms of TG in vivo and the dietary habits of the Chinese diabetic population further support the potential use of postprandial blood lipid measurements for Chinese CHD patients with DM. Furthermore, our study indicated that serum TG and RLP-C concentrations are closely correlated, with the relationship appearing to be much stronger postprandial. There is no direct evidence to support TG involvement in atherosclerosis. The current study suggests that TG could serve as a marker reflective of TRL and/or RLP-C levels [19]. In contrast to LDL, RLP, which is relatively enriched with cholesterol, can be directly phagocytised by macrophages to form foam cells without having to undergo oxidation [20]. Previous studies have suggest that non-fasting RLP-C contributes to the increase of death rate, as well as ASCVD specifically [21–23].

As renal complications from DM contribute to adverse prognoses for patients with CHD as well as those with ASCVD [25, 26], we utilised UACR as a sensitive indicator of early renal damage [24] and observed that fasting serum TG and RLP-C concentrations were positively associated with UACR, with the correlation stronger under postprandial conditions compared with fasting conditions. Furthermore, we demonstrated that the systemic inflammatory markers TNF-α and IL-6 were significantly increased at 4-h postprandial, and that both markers were also significantly correlated with both TRL and UACR concentrations under postprandial conditions. These results support previous studies that have reported correlations between increased postprandial TRLs and induced systemic inflammation [27, 28]. However, our study is the first to indicate that elevated postprandial TRLs also increase systemic inflammation in patients with diabetes and SCAD. This is significant as it has been shown that serum IL-6 potentially induces early renal damage by altering glomerular basement membrane permeability, thereby promoting mesangial cell proliferation and increasing fibrin expression [29]. Moreover, TNF α has been shown to be directly involved in renal cytotoxicity by promoting vascular smooth cells apoptosis and necrosis [30]. As such, our results indicate that the observed increase of UACR in patients with DM and SCAD could be partly attributed to systemic inflammation induced by increased postprandial TRLs. Importantly, we did not observe any significant correlations between TC, LDL-C, HDL-C, non-HDL-C levels, serum inflammation cytokines and UACR under either fasting or postprandial conditions. Current guidelines for both lipid management and chronic kidney disease recommended using LDL-C concentration as the primary marker for assessing ASCVD risk [6, 31]. However, the current study shows that TRLs should be considered when assessing and treating patients with DM and SCAD.

### Comparisons with other studies and what does the current work add to the existing knowledge

We successfully demonstrated that serum TG and RLP-C concentrations 4-h after daily Chinese breakfast were much higher than those at fasting. Moreover, strong positive correlations we observed between TG, RLP-C, systemic inflammation cytokines and UACR in patients with DM and SCAD, especially under postprandial conditions.

### Study strengths and limitations

As a pilot study, the strength of this study mainly lies in the interesting and novel findings. However, this study has one significant limitation. The small sample size and observational nature of the study means that the association between TG and/or RLP-C, systemic inflammation and UACR could only be inferred based on correlation analyses. As such, larger-scale prospective trials should be considered in the future in order to establish potential causality.

## Conclusions

Overall, the present study has demonstrated that it is feasible to accurately detect postprandial lipid profiles after daily Chinese breakfast in patients with DM and SCAD. Postprandial lipid measurement could prevent hypoglycaemic events, more importantly, the postprandial TRLs are probably involved in early renal injury through the induction of systemic inflammation. In the future, routine testing of postprandial lipid profile in patients with DM and SCAD should be recommended in clinical practice.

## Supplementary Information

**Additional file 1.** Original Data. Data set supporting the conclusions of this article.

## Data Availability

All data generated or analysed during this study are included in this published article [and its supplementary information files].

## Abbreviations

CVD: Cardiovascular disease
HTG: Hypertriglyceridemia
SCAD: Stable coronary artery disease
DM: Diabetes mellitus
TG: Triglyceride
CHD: Coronary heart disease
T2D: Type 2 diabetes
LDL-C: Low-density lipoprotein cholesterol
HDL-C: High-density lipoprotein cholesterol
sdLDL-C: Small, dense low-density lipoprotein
CM: Chylomicron
VLDL: Very low density lipoprotein
TRLs: Triglyceride-rich lipoproteins
RLPs: Remnant-like particles
RLP-C: Remnant lipoprotein-cholesterol
non-HDL: Non-high-dense lipoprotein-cholesterol
UACR: Ratio of urinary albumin to creatinine
TRLs: Triglyceride-Rich Lipoproteins
IL-6: Interleukin-6
TNF-α: Tumour necrosis factor-α
Cr: Creatinine
HbA1c: Glycated haemoglobin
ELISA: Enzyme-linked immunosorbent assay
FBG: Fasting blood glucose
eGFR: Estimated glomerular filtration rate
NYHA: New York Heart Association
ASCVD: Atherosclerotic cardiovascular disease
AST: Aspartate transaminase
ALT: Alanine transaminase
FT4: Free thyroxine
FT3: Triiodothyronine
TSH: Thyroid-stimulating hormone

## References

[1] Bertoluci MC, Rocha VZ (2017). Cardiovascular risk assessment in patients with diabetes. Diabetol Metab Syndr.

[2] González-Pérez A, Saez M, Vizcaya D, Lind M, Garcia Rodriguez L (2021). Incidence and risk factors for mortality and end-stage renal disease in people with type 2 diabetes and diabetic kidney disease: a population-based cohort study in the UK. BMJ Open Diabetes Res Care.

[3] Thambiah SC, Lai LC (2021). Diabetic dyslipidaemia. Pract Lab Med.

[4] Arca M, Veronesi C, D'Erasmo L, Borghi C, Colivicchi F, De Ferrari GM, Desideri G, Pontremoli R, Temporelli PL, Perrone V (2020). Association of hypertriglyceridemia with all-cause mortality and atherosclerotic cardiovascular events in a low-risk italian population: the TG-REAL retrospective cohort analysis. J Am Heart Assoc.

[5] Saeed A, Feofanova EV, Yu B, Sun W, Virani SS, Nambi V, Coresh J, Guild CS, Boerwinkle E, Ballantyne CM, Hoogeveen RC (2018). Remnant-like particle cholesterol, low-density lipoprotein triglycerides, and incident cardiovascular disease. J Am Coll Cardiol.

[6] Grundy SM, Stone NJ, Bailey AL, Beam C, Birtcher KK, Blumenthal RS, Braun LT, de Ferranti S, Faiella-Tommasino J, Forman DE (2019). 2018 AHA/ACC/AACVPR/AAPA/ABC/ACPM/ADA/AGS/APhA/ASPC/NLA/PCNA Guideline on the management of blood cholesterol: a report of the american college of cardiology/american heart association task force on clinical practice guidelines. Circulation.

[7] Langsted A, Nordestgaard BG (2011). Nonfasting lipids, lipoproteins, and apolipoproteins in individuals with and without diabetes: 58 434 individuals from the Copenhagen General Population Study. Clin Chem.

[8] Bansal S, Buring JE, Rifai N, Mora S, Sacks FM, Ridker PM (2007). Fasting compared with nonfasting triglycerides and risk of cardiovascular events in women. JAMA.

[9] Ceriello A, Assaloni R, Da Ros R, Maier A, Piconi L, Quagliaro L, Esposito K, Giugliano D (2005). Effect of atorvastatin and irbesartan, alone and in combination, on postprandial endothelial dysfunction, oxidative stress, and inflammation in type 2 diabetic patients. Circulation.

[10] Libby P, Ridker PM, Hansson GK (2011). Progress and challenges in translating the biology of atherosclerosis. Nature.

[11] Plowman TJ, Shah MH, Fernandez E, Christensen H, Aiges M, Ramana KV. Role of innate immune and inflammatory responses in the development of secondary diabetic complications. Curr Mol Med. 2022. 10.2174/1566524023666220922114701. 10.2174/1566524023666220922114701 36154569

[12] Task Force M, Montalescot G, Sechtem U, Achenbach S, Andreotti F, Arden C, Budaj A, Bugiardini R, Crea F, Cuisset T (2013). 2013 ESC guidelines on the management of stable coronary artery disease: the Task Force on the management of stable coronary artery disease of the European Society of Cardiology. Eur Heart J.

[13] Chinese Society of Cardiology, Editorial Committee of Chinese Journal of Cardiology. Guidelines for diagnosis and treatment of chronic stable angina. Zhonghua Xin Xue Guan Bing Za Zhi 2007, 35:195–206. 17582280

[14] Nordestgaard BG, Langsted A, Mora S, Kolovou G, Baum H, Bruckert E, Watts GF, Sypniewska G, Wiklund O, Boren J (2016). Fasting is not routinely required for determination of a lipid profile: clinical and laboratory implications including flagging at desirable concentration cut-points-a joint consensus statement from the European Atherosclerosis Society and European Federation of Clinical Chemistry and Laboratory Medicine. Eur Heart J.

[15] Liosis S, Hochadel M, Darius H, Behrens S, Mudra H, Lauer B, Elsässer A, Gitt AK, Zahn R, Zeymer U (2019). Effect of renal insufficiency and diabetes mellitus on in-hospital mortality after acute coronary syndromes treated with primary PCI. Results from the ALKK PCI Registry. Int J Cardiol.

[16] Kelley GA, Kelley KS, Roberts S, Haskell W (2011). Efficacy of aerobic exercise and a prudent diet for improving selected lipids and lipoproteins in adults: a meta-analysis of randomized controlled trials. BMC Med.

[17] Wilson SM, Maes AP, Yeoman CJ, Walk ST, Miles MP (2021). Determinants of the postprandial triglyceride response to a high-fat meal in healthy overweight and obese adults. Lipids Health Dis.

[18] Xu J, Chen YQ, Zhao SP, Liu L (2019). Determination of optimal cut-off points after a high-fat meal corresponding to fasting elevations of triglyceride and remnant cholesterol in Chinese subjects. Lipids Health Dis.

[19] Hayashi T, Ai M, Goto S, Nakamura M, Nagaike H, Suzuki R, Abe Y, Ohta M, Ito Y, Hirano T. Circadian Rhythm of Subspecies of Low-Density Lipoprotein-Cholesterol and High-Density Lipoprotein-Cholesterol in Healthy Subjects and Patients with Type 2 Diabetes. J Atheroscler Thromb. 2023;30(1):3–14. 10.5551/jat.63383 PMC9899707 35249932

[20] Nordestgaard BG, Varbo A (2014). Triglycerides and cardiovascular disease. Lancet.

[21] Nordestgaard BG, Tybjaerg-Hansen A, Lewis B (1992). Influx in vivo of low density, intermediate density, and very low density lipoproteins into aortic intimas of genetically hyperlipidemic rabbits. Roles of plasma concentrations, extent of aortic lesion, and lipoprotein particle size as determinants. Arterioscler Thromb.

[22] Fukushima H, Sugiyama S, Honda O, Koide S, Nakamura S, Sakamoto T, Yoshimura M, Ogawa H, Fujioka D, Kugiyama K (2004). Prognostic value of remnant-like lipoprotein particle levels in patients with coronary artery disease and type II diabetes mellitus. J Am Coll Cardiol.

[23] Varbo A, Benn M, Tybjaerg-Hansen A, Jorgensen AB, Frikke-Schmidt R, Nordestgaard BG (2013). Remnant cholesterol as a causal risk factor for ischemic heart disease. J Am Coll Cardiol.

[24] Keane WF, Eknoyan G (1999). Proteinuria, albuminuria, risk, assessment, detection, elimination (PARADE): a position paper of the National Kidney Foundation. Am J Kidney Dis.

[25] Shah SJ, Lam CSP, Svedlund S, Saraste A, Hage C, Tan RS, Beussink-Nelson L, Ljung Faxén U, Fermer ML, Broberg MA (2018). Prevalence and correlates of coronary microvascular dysfunction in heart failure with preserved ejection fraction: PROMIS-HFpEF. Eur Heart J.

[26] Zand Parsa AF, Ghadirian L, Rajabzadeh Kanafi S, Moradi Farsani E (2013). Positive correlation between microalbuminuria and severity of coronary artery stenosis in patients with type 2 diabetes mellitus. Acta Med Iran.

[27] Vazquez-Madrigal C, Lopez S, Grao-Cruces E, Millan-Linares MC, Rodriguez-Martin NM, Martin ME, Alba G, Santa-Maria C, Bermudez B, Montserrat de la Paz S (2020). Dietary fatty acids in postprandial triglyceride-rich lipoproteins modulate human monocyte-derived dendritic cell maturation and activation. Nutrients.

[28] Padro T, Muñoz-Garcia N, Badimon L (2021). The role of triglycerides in the origin and progression of atherosclerosis. Clin Investig Arterioscler.

[29] Navarro-Gonzalez JF, Mora-Fernandez C (2011). Muros de Fuentes M, Garcia-Perez J: Inflammatory molecules and pathways in the pathogenesis of diabetic nephropathy. Nat Rev Nephrol.

[30] Boyle JJ, Weissberg PL, Bennett MR (2003). Tumor necrosis factor-alpha promotes macrophage-induced vascular smooth muscle cell apoptosis by direct and autocrine mechanisms. Arterioscler Thromb Vasc Biol.

[31] Wanner C, Tonelli M (2014). KDIGO Clinical Practice Guideline for Lipid Management in CKD: summary of recommendation statements and clinical approach to the patient. Kidney Int.

